# Shear Stress in Schlemm’s Canal as a Sensor of Intraocular Pressure

**DOI:** 10.1038/s41598-020-62730-4

**Published:** 2020-04-02

**Authors:** Fiona McDonnell, Kristin M. Perkumas, Nicole E. Ashpole, Joan Kalnitsky, Joseph M. Sherwood, Darryl R. Overby, W. Daniel Stamer

**Affiliations:** 1 Duke Eye Center, Durham, North Carolina USA; 2 Bioengineering, Imperial College London, London, UK; 3 Biomedical Engineering, Duke University, Durham, USA

**Keywords:** Physiology, Eye diseases

## Abstract

Elevated intraocular pressure (IOP) narrows Schlemm’s canal (SC), theoretically increasing luminal shear stress. Using engineered adenoviruses containing a functional fragment of the shear-responsive endothelial nitric oxide synthase (eNOS) promoter, we tested effects of shear stress and elevated flow rate on reporter expression *in vitro* and *ex vivo*. Cultured human umbilical vein endothelial cells (HUVECs) and SC cells were transduced with adenovirus containing eNOS promoter driving secreted alkaline phosphatase (SEAP) or green fluorescent protein (GFP) and subjected to shear stress. In parallel, human anterior segments were perfused under controlled flow. After delivering adenoviruses to the SC lumen by retroperfusion, the flow rate in one anterior segment of pair was increased to double pressure. In response to high shear stress, HUVECs and SC cells expressed more SEAP and GFP than control. Similarly, human anterior segments perfused at higher flow rates released significantly more nitrites and SEAP into perfusion effluent, and SC cells expressed increased GFP near collector channel ostia compared to control. These data establish that engineered adenoviruses have the capacity to quantify and localize shear stress experienced by endothelial cells. This is the first *in situ* demonstration of shear-mediated SC mechanobiology as a key IOP-sensing mechanism necessary for IOP homeostasis.

## Introduction

Glaucoma is an optic neuropathy, resulting from progressive retinal ganglion cell death^1^. This disease affects more than 60 million people worldwide, and is the leading cause of irreversible blindness. The primary risk factor for glaucoma is elevated intraocular pressure (IOP)^2,3^, which is caused by increased outflow resistance to aqueous humor through the conventional outflow pathway. Resistance in this pathway is generated principally in the juxtacanalicular region (JCT), where the trabecular meshwork (TM) and Schlemm’s canal (SC) cells interact^4,5^. After passing through the JCT, aqueous humor enters the SC lumen, and exits via collector channels, aqueous veins and the  intrascleral venous plexus.

Schlemm’s canal is a circular, collapsible vessel lined with endothelial cells that are shear stress responsive^6^. Increasing IOP, as seen in glaucoma, causes a decrease in SC lumen diameter and correspondingly stretches the TM tissue^7,8^. Numerical models indicate that SC shear stress and TM stretch are sensitive to resistance in the JCT region, suggesting that these are mechanical responses involved in IOP homeostasis^9^. Resistance in the conventional outflow pathway is regulated to maintain IOP homeostasis^10,11^ and the likely mechanosensors involved in this process are the SC and TM cells. Within the SC, the circumferential flow of aqueous humor towards collector channel ostia applies shear stress to the endothelial cells lining the lumen. Significantly, the levels of shear stress in SC have been calculated to be comparable to those seen in large arteries^9,12^. When tested in culture, elevated shear stress across SC cell monolayers results in increased nitric oxide (NO) production by endothelial nitric oxide synthase (eNOS)^6^. Within the conventional outflow pathway, perfusing with NO donors has been shown to be an effective way of increasing outflow facility and lowering IOP in both mouse^13^ and human eyes^14^.

As with SC, the primary source of NO produced by endothelial cells in other tissues is eNOS. The expression of eNOS is regulated by a variety of stimuli, including hypoxia and shear stress^15,16^, which is the frictional force that blood imparts upon endothelial cells as it moves through vessels. Importantly, regions of blood vessels that experience high levels of shear stress are protected from the development of atherosclerotic lesions compared to regions of low shear stress^17^. This protection can be attributed to the antithrombogenic, vasodilatory and antiproliferative properties of NO produced by eNOS^18^.

The expression and function of eNOS is important for vascular homeostasis, serving as a highly sensitive system to maintain appropriate blood flow to tissue beds. Thus, studies have shown eNOS is important for remodelling of vessel walls in response to alterations in flow or internal vessel pressure^19–21^. Mice engineered to overexpress eNOS demonstrate attenuation of cardiac and pulmonary dysfunction in response to induced congestive heart failure^22^, and improved cerebral vascular function^23^. Whereas when eNOS is ablated in mice, cardiovascular homeostasis is disrupted and mice are hypertensive. Not surprisingly, altered expression of eNOS is a defining feature of a general class of diseases termed “endothelial dysfunction”, implicated in a number of cardiovascular diseases and recently ocular hypertension. Accordingly, genetic polymorphisms within the eNOS gene have been linked to ocular hypertension and primary open angle glaucoma in different populations^24–32^. In the eye, eNOS overexpressing mice have decreased IOP and increased pressure-dependent outflow facility^33^. Thus, understanding the molecular regulation of eNOS gene expression is an important research area that appears to drive a number of disease processes, including elevated IOP in glaucoma. In particular, we hypothesize that the shear stress response of SC cells involves NO production by eNOS to comprise a critical mechanosensitive feedback signal that is partly responsible for homeostatic normalization of IOP.

In the present study, we engineered, constructed and tested two novel reporter vectors containing the shear stress responsive element of the eNOS promoter to monitor the distribution and quantify the magnitude of shear stress in Schlemm’s canal.

## Results

To determine if shear stress in SC could detect changes in IOP, we first designed and engineered two adenovirus reporters and validated them in cell culture models using known levels of applied shear stress. We then introduced these adenoviruses into a physiological model of outflow facility, the anterior segment perfusion model, to determine if we could utilise these reporters, and therefore shear stress, to detect changes in IOP induced by an increased flow rate.

### Characterization of shear stress-mediated changes in protein reporter expression in two endothelial cell types

**Figure 1:**
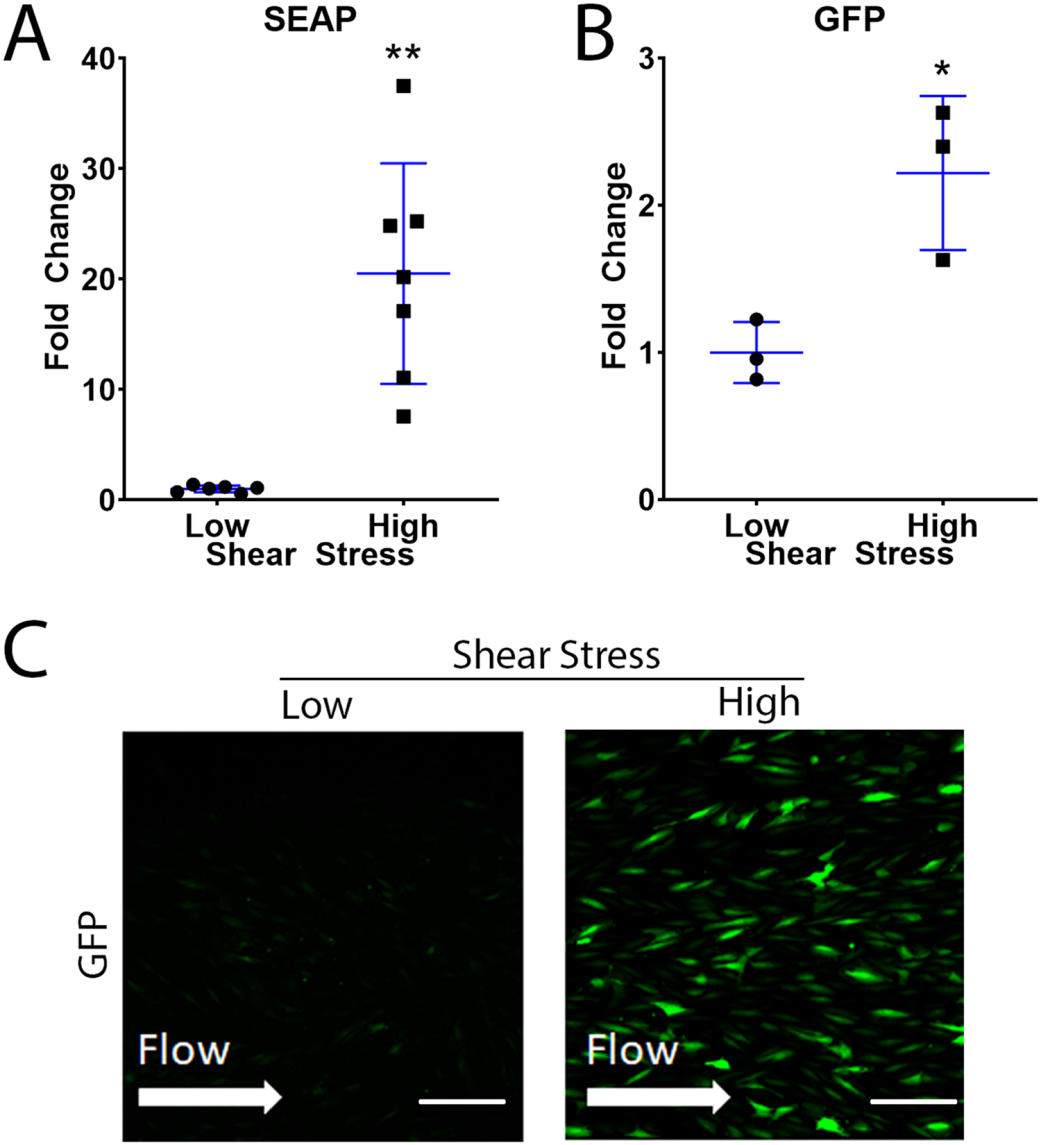
Shear stress-mediated changes in expression of SEAP and GFP by HUVECs. HUVECs were transduced with either eNOS-SEAP or eNOS-GFP adenovirus and subjected to low or high shear stress for 24 hours. (**A**) SEAP concentration in conditioned media was assessed by a chemiluminescent SEAP assay. Cellular GFP expression was measured using (**B**) flow cytometry and (**C**) confocal microscopy. Low shear = 0.1–1 dynes/cm^2^ and high shear = 10–12.5 dynes/cm^2^. Scale bar–50 µm. *P < 0.05, **P < 0.01.

The signal-to-noise ratio of two different reporter proteins driven by the eNOS promoter were evaluated. First, HUVECs were transduced with eNOS-SEAP or eNOS-GFP adenovirus and subjected to shear stress. Results show an increased secretion of SEAP into conditioned media (20.5 ± 10.0 fold change, P = 0.002) at high shear stress (n = 7) compared to low shear stress (n = 6) (10–12.5 vs. 0.1–1 dynes/cm^2^) (Fig. 1A). GFP expression by transduced cells was evaluated in two ways, by immunofluorescence microscopy and flow cytometry. Flow cytometry demonstrated an increase in GFP expression by HUVECs subjected high shear stress (2.2 ± 0.5 fold change, P = 0.04) (Fig. 1B). Immunofluorescence microscopy was consistent with other methods, showing qualitatively increased levels of GFP expression by cells exposed to 10–12.5 dynes/cm^2^ compared to 0.1–1 dynes/cm^2^ (Fig. 1C).

**Figure 2:**
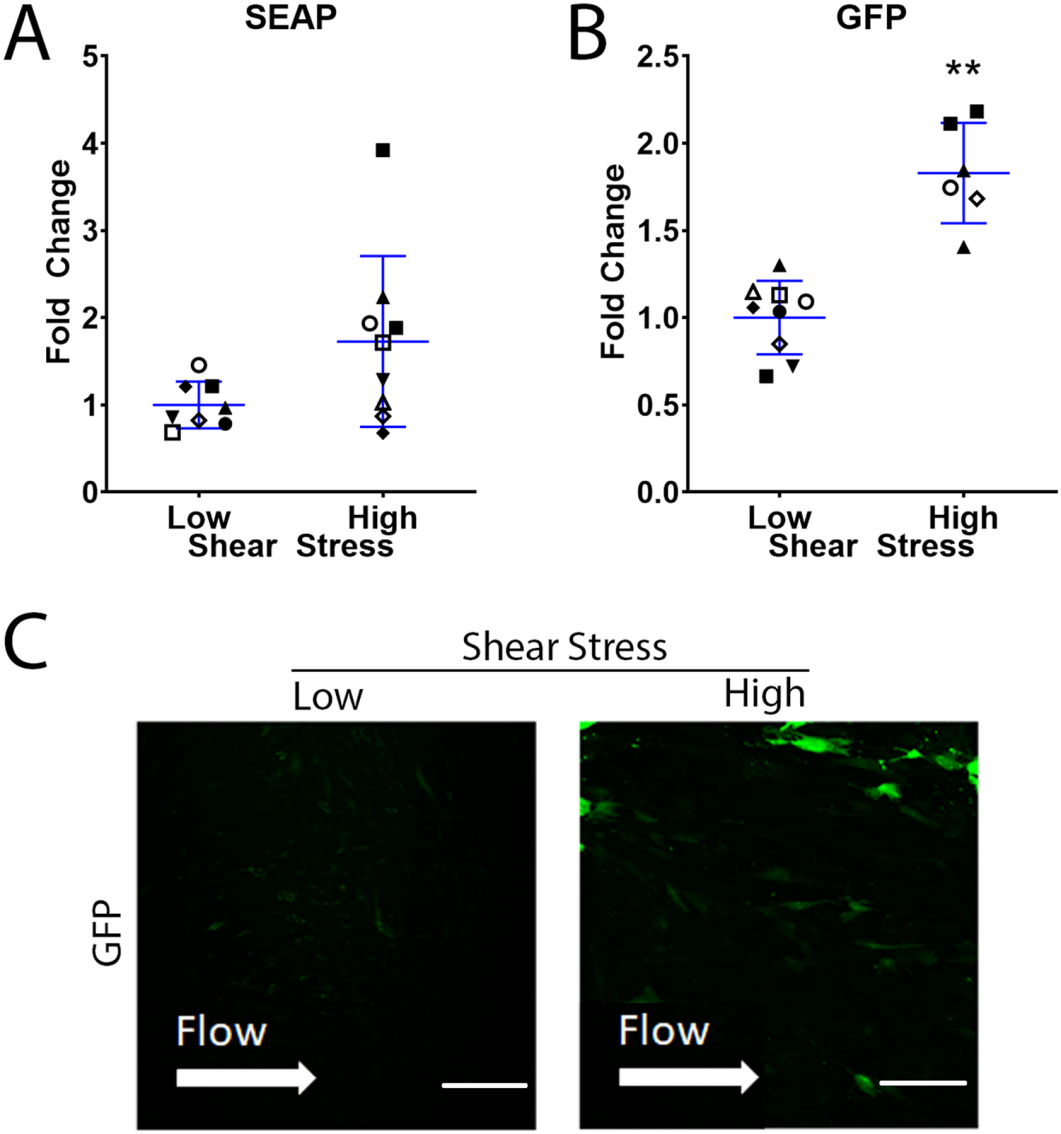
Shear stress-mediated changes in expression of SEAP and GFP by SC cells. SC cells were transduced with either eNOS-SEAP or eNOS-GFP adenovirus and subjected to low or high shear stress for 24 hours. (**A**) SEAP concentration in conditioned media was assessed by a chemiluminescent SEAP assay. Cellular GFP expression was measured using (**B**) flow cytometry and (**C**) confocal microscopy. Low shear = 0.1–1 dynes/cm^2^ and high shear = 10–12.5 dynes/cm^2^. Scale bar–50 µm. *P < 0.05., **P < 0.01.

Media collected from SC cells that were subjected to shear stress was also assessed for SEAP concentration. SC cells transduced with eNOS-SEAP adenovirus secreted significantly more SEAP into conditioned media at high shear stress (n = 9) in comparison to low shear stress (n = 8) (1.7 ± 1.0 fold change, P = 0.06) (Fig. 2A). The two methods to evaluate GFP expression by SC cells showed comparable results: Flow cytometry demonstrated that cells exposed to 10–12.5 dynes/cm^2^ (n = 6) expressed 1.8 ± 0.3, p = 0.0002 fold higher levels of GFP compared to cells experiencing 0.1–1 dynes/cm^2^ (n = 9) (Fig. 2B). Lastly, GFP expression as visualized by immunofluorescence microscopy was qualitatively elevated at high shear stress (Fig. 2C).

Results from both flow cytometry and immunofluorescence microscopy demonstrate dramatic differences in transduction efficiency when comparing HUVECs and SC cells (93 ± 1% in HUVECs vs. 14 ± 11% in SC cells), which affected the signal to noise ratio of the different assays. However, both cell types had relatively high cell viability when transduced under both low and high shear conditions; in HUVEC cells transduced with our eNOS-GFP vector, we found that cell viability was 75% and 58% at low and high shear respectively. SC cells transduced with eNOS-GFP had cell viability of 93% and 89% at low and high shear stress respectively.

### Intrachamber pressure elevation triggers increased levels of nitric oxide and reporter proteins in Schlemm’s canal

**Figure 3:**
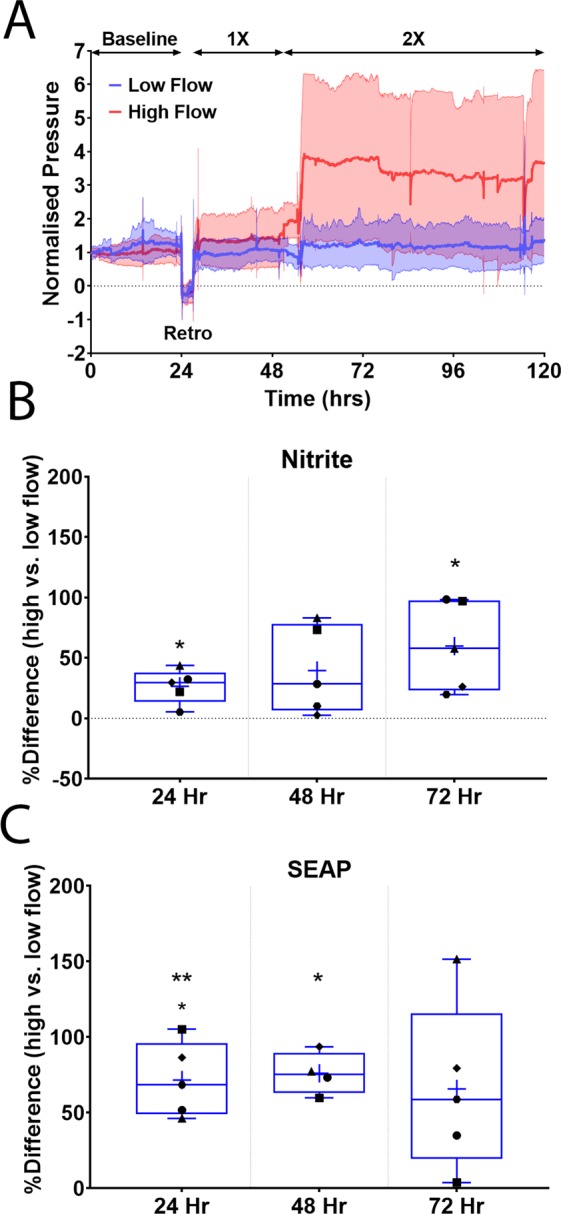
Human anterior segment pairs retroperfused with adenovirus reporters. Paired human anterior segments were perfused at a constant flow rate of 2.5 µl/min to achieve stable baseline intrachamber pressures. Following this, a cocktail of both eNOS-SEAP and eNOS-GFP viruses was retroperfused into both anterior segments. The flow rate in one segment was then increased to 5 µl/min. Effluent media was then collected every 24 hours after the increase in flow rate for analyses. (**A**) Perfusion trace showing manipulations throughout perfusion, solid line represents mean, shaded areas show standard deviation. (**B**) Nitrite concentration by nitrite quantification assay and (**C**) SEAP concentration was measured using a chemiluminescent SEAP assay. Indicated times are post-increase in flow rate. Samples were normalized to volume. n = 5, *P < 0.05, **P < 0.01, 1× = 2.5 µl/min, 2× = 5 µl/min flow rates.

**Figure 4:**
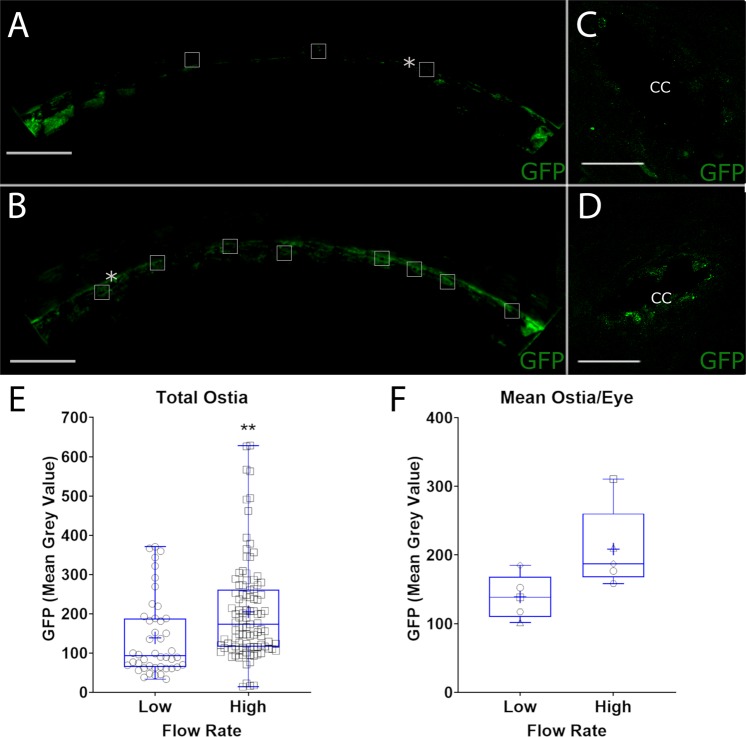
Shear stress-responsive GFP expression by Schlemm’s canal at elevated intrachamber pressure. Human anterior segment pairs transduced with adenovirus containing shear stress responsive GFP reporter were perfused at two flow rates, and thus normal and elevated intrachamber pressures. Shown are representative immunofluorescence images of GFP expression by SC cells located on the outer wall at low flow rate in one eye quadrant at low (**A**) and at high flow rates (**B**). Increased GFP expression is particularly evident by SC cells located at collector channel ostia (boxes in **A,B**, magnified in **C,D**). Panel E shows quantification of GFP fluorescence of all collector channels identified, while panel F shows combined data of individual anterior segments. Each data point represents mean ostia data from each anterior segment. *Representative collector channels shown in (**C,D**), respectively. Scale Bars (**A,B**)–500 µm, (**C,D**)–50 µm **P < 0.01.

To examine utility of viruses in a preparation with intact conventional outflow tract, paired human anterior segments were perfused at 2.5 µl/min for 24–48 hours until a stable baseline pressure was achieved (Fig. 3A). Anterior segment pairs were then transduced with an eNOS-SEAP and eNOS-GFP adenovirus cocktail using a retroperfusion technique. Immediately afterwards, the forward perfusion was restarted at 2.5 µl/min. After 24 hours, perfusion of one anterior segment remained at a flow rate of 2.5 μl/min while the flow rate into the contralateral segment was increased to approximately double the pressure. Effluent was collected over time of perfusion to assess SEAP secretion and nitric oxide concentration. Nitric oxide levels as reflected by nitrite concentration in effluent were significantly elevated in anterior segments subjected to the higher flow rate throughout the experiment (Table 1, Fig. 3B). Likewise, SEAP concentration in the effluent from anterior segments perfused at the high flow rate was significantly elevated (Table 1, Fig. 3C). To assess regional differences in shear stress experienced by SC cells we imaged GFP expression 360 degrees around the outer wall of SC by immunofluorescence microscopy. We observed that GFP expression was consistently more robust in SC of anterior segments exposed to the higher flow rates (Fig. 4B) compared to the low flow rates (Fig. 4A). Interestingly, we noticed that GFP expression was particularly elevated at collector channel ostia of segments perfused at the higher flow rate (Fig. 4D) compared to the low flow rate (Fig. 4C). Unbiased quantification of GFP expression at collector channel ostia demonstrated a significant 61% increase in GFP expression at the higher flow rate (Fig. 4E (P = 0.001), 4 F (P = 0.06)).

**Table 1:** Percent difference in nitrite concentration and SEAP concentration at high flow compared to low flow.

Post Flow Rate Increase	% Difference in Nitrite Concentration	P-Value	% Difference in SEAP Concentration	P-Value
24 Hr	27% ± 14%	0.01	72% ± 24%	0.003
48 Hr	40% ± 37%	0.07	76% ± 14%	0.002
72 Hr	60% ± 37%	0.02	66% ± 56%	0.06

To maximize use of donated human eyes, anterior segments pairs where only with one segment established a stable baseline pressure (Supplemental Fig. 2A) were subject to a different protocol. Unpaired segments were retroperfused with a cocktail containing both eNOS-SEAP and eNOS-GFP adenoviruses, and forward perfusion was restarted. Flow rate was then increased in segments to 5 µl/min for 24 hours. All the while, perfusion media effluent was collected as it exited the eye over time and assayed for SEAP secretion and nitric oxide content (Supplemental Fig. 2B,C). Both nitrite and SEAP levels were elevated (1.7 ± 1.5, P = 0.13) and (1.7 ± 0.4, P = 0.05), respectively at the higher flow rate compared to baseline (Supplemental Fig. 2B,C). Adenovirus transduction of SC was confirmed by immunofluorescence analysis of GFP expression by outer wall cells (Supplemental Fig. 3).

## Discussion

In this study, we tested two engineered adenoviruses containing reporters for their ability to quantify and localize shear stress in Schlemm’s canal, hypothesized to be a critical part of a feedback system that regulates IOP. We successfully quantified shear stress responses by two different endothelial cell types in culture and conventional outflow endothelia *in situ* using constructs containing a fragment of the eNOS promoter driving SEAP or GFP protein expression. We found that flow-induced increases in intrachamber pressure increased SEAP and GFP expression within/by SC. Importantly, we experimentally validated *in silico* models of shear stress in SC showing pressure-induced increases in both GFP and SEAP expression, particularly by SC cells located near collector channel ostia. Taken together, this is the first demonstration of SC cells sensing shear stress as predicted by numerical modelling.

Since these were novel reporter constructs, we first wanted to determine whether they were responsive to shear stress and determine magnitude of responses in a controlled fashion. We used two shear-responsive endothelial cell types to characterize the effectiveness of the adenovirus reporter constructs in quantifying and identifying acute levels of shear stress (<24 hours). We exposed cells to two different, physiologically relevant levels of shear stress; using 0.1–1 dynes/cm^2^ to provide a sufficient level of media turnover for cell viability, but essentially no mechanical stress on the cells, and 10–12.5 dynes/cm^2^ which has been determined as a level of shear reached in SC at increased levels of intraocular pressure^9,12^. SEAP secretion by cells exposed to elevated shear stress was increased in HUVECs and SC cells. Finally, using two different methods, GFP expression was elevated at 10–12.5 dynes/cm^2^ shear stress in HUVECs and SC cells compared to 0.1–1 dynes/cm^2^. We also found that adenovirus was more efficient at transducing HUVECs than SC cells, producing more consistent data with elevated shear stress; this may be because SC cell strains have previously been shown to be slower at producing NO under shear stress than HUVECs^6^. Regardless, these data demonstrated the ability of the adenovirus reporters to effectively quantify and identify acute shear stress in a controlled system.

In the present study, we used an anterior chamber perfusion model to investigate shear stress sensation in Schlemm’s canal. In particular, we were interested in whether shear stress on SC endothelium might act as sensor of elevated IOP *in vivo*. Within the context of this study, we investigated the efferent arm of the hypothesized feedback loop – the ability of the SC cells to sense a change in shear stress and increase eNOS activity. The most likely cause of such IOP elevation is an increase in the resistance to outflow in the vicinity of the JCT and inner wall of SC. As there is no clean way to elevate this resistance experimentally, we instead used the established approach of doubling the flow rate. The shear stress in SC is predominantly determined by the flow through it and its height. Due to conservation of mass, the flow through SC increases directly with the flow through the system, so doubling the flow rate would approximately double the SC shear stress. However, doubling flow rate also doubles the pressure drop across the JCT and inner wall of SC, which would approximately halve the SC height. As SC shear stress is inversely proportional to the square of its height, the combination of effects could be expected to increase shear stress by a factor of approximately 8. By comparison with our previous model^9^, we estimate that this increase in shear stress is comparable to what would occur if resistance to flow adjacent to the inner wall increased approximately 3-fold, elevating the intraocular pressure from 15 mmHg to 23 mmHg.

To directly test whether an increased flow rate, and therefore, elevated pressure increases shear stress in SC, we monitored NO production using nitrite levels as an indirect indicator. In both of our experimental paradigms, nitrite levels were higher in segments with the higher flow rate. This is integral part of IOP homeostasis, as NO has been shown to lower IOP and increase facility in mice^13^ and humans^34,35^. Schneemann *et al*. also investigated the effect of increased IOP on NO production in an anterior segment perfusion model using constant pressure^36^. Similar to our results with a constant flow system, they found an increase in both flow rate and NO production at 25 mmHg compared to 10 mmHg. However, this early study was not able to quantify or localize regions of elevated shear stress.

Like nitrite, we were able to monitor our secreted reporter, SEAP in perfusion effluent. In both paired and unpaired anterior segments, we found SEAP secretion increased during the 24 hr period following flow rate increase, indicating the eNOS promoter was executing an acute response to shear stress. This is similar to our *in vitro* studies in both HUVECs and SC endothelial cells showing an acute response to applied shear stress by increasing SEAP secretion at 10–12.5 dynes/cm^2^. Interestingly, in paired anterior segments the level of secretion was steadily maintained throughout the perfusion (up to 72 hours post transduction) indicating a sustained effect of eNOS promoter activity at an increased flow rate. Matching this trend, the nitrite concentrations increased at each time point indicating that shear stress was chronically elevated during this time period. Most important for these experiments was that nitrite levels were used as a positive control to indicate that the increase in flow rate was imparting significant shear stress on the cells.

These data are consistent with models of IOP homeostasis indicating that shear stress is a critical regulator of IOP within Schlemm’s canal^9^. Upon IOP elevation, eNOS is activated by shear stress and upregulated within SC endothelial cells increasing production of NO and leading to a decrease in IOP. This can happen through both acute and chronic mechanisms; for the purposes of this study, we focused on the effects of chronic stress within the conventional outflow pathway. Mechanistically, chronic shear stress regulates eNOS activity through two pathways; regulation of eNOS transcription and control of eNOS mRNA stability. In response to the onset of shear stress, transcription of eNOS is transiently upregulated by NFkB activation and p50/p65 binding to a GAGACC sequence in the eNOS promoter at the shear stress responsive element (SSRE)^37^. While c-Src appears to direct both eNOS transcription through activity of ERK1/2 and IkKa and activity of eNOS via its phosphorylation, the mechanism by which c-Src works to stabilize mRNA is unclear^38,39^. The chronic impact of shear stress (>24 hrs) involves increased production of transforming growth factor-β (TGFβ) and subsequent activation of Krüppel-like factor-2 (KLF2), which robustly promotes eNOS gene transcription as part of the cAMP responsive binding protein/p300 complex binding between nucleotides −730 to −660 of the eNOS promoter^39^. Due to these findings, our earliest time point was 24 hours after induction of shear stress.

To localize sites of IOP-mediated elevation in shear stress, we monitored our GFP reporter. Data show that the adenoviruses successfully reached the SC lumen using our retroperfusion technique and that shear stress at the level of SC was elevated. Additionally, we identified specific locations of increased shear stress along the outer wall of SC. Significantly, we noticed an increase in GFP expression specifically surrounding collector channel ostia, consistent with computer models showing increased shear stress at these locations^9,12^ and with experimental data showing better alignment of SC cells at ostia, which is also an indicator of shear stress^40^. Further, Hann *et al*. demonstrated that collector channel ostia are increased by elevated IOP making them more identifiable^41^, a process which supports our hypothesis of outflow cells regulating IOP in response to shear stress. Using SEAP or nitrite as a readout in the anterior segment perfusions, we cannot separate shear stress responses of SC cells from endothelial cells in the distal portion of the outflow tract. However, given the GFP labelling of SC and collector channels shown in Fig. 4, it is clear that a portion of nitrite and SEAP are produced by SC cells. However, the distal vessels are difficult to study in a controlled fashion. Theoretically, only vessels that are continuous with flow pathways directly connected to conventional outflow will be transduced due to the induced pressure gradient at the level of the anterior chamber. However, monitoring fluorescence in these distal vessels using techniques developed for canalograms would be useful for future studies to determine GFP expressing within the distal outflow pathway^42,43^. Interestingly, we also observed differential levels of GFP expression circumferentially around SC of segments at either flow rate, suggesting gradients of shear stress in SC and/or differences in access of adenovirus to SC cells^44–46^.

## Conclusions

Numerical models predict that levels of shear stress in SC at elevated IOPs approach that experienced by large vessel endothelial cells, and that the highest level of shear stress occurs at collector channel ostia. This is part of a larger process involved in IOP homeostasis through shear stress induced nitric oxide production. The present study experimentally confirms these theoretical predictions with empirical data in a relevant physiological model of the human conventional outflow pathway. This is the first such study to demonstrate this important shear-mediated response within the outflow pathway.

## Materials and Methods

### Plasmid preparation

A 1037 bp fragment of the eNOS promoter was isolated from human genomic DNA^47^. The 5′ primer added a HindIII restriction site (5′-AAA AGC TTC CGT TTC TTT CTT AAA CT-3′) and the 3′ primer added an EcoRI restriction site (5′-GAA TTC GTT ACT GTG CGT CCA CTC T-3′) to the ends. The DNA fragment was then inserted into the pEGFP-N1 or pSEAP-basic plasmid. DNA containing the eNOS promoter plus GFP polyA tail or SEAP polyA tail was subsequently cloned into the pENTR1A plasmid to enable generation of adenovirus (Life Technologies Virapower Adenovirus Expression System). Thus, the pENTR1A clone with the eNOS promoter driving either GFP or SEAP was recombined with the pAd/PL-DEST^TM^ vector. The resulting plasmid was then Pac1 digested and transfected into 293 A cells that contain the E1 region that permits the production of replication incompetent adenovirus. Adenovirus was harvested from the lysed cells and media, then purified by centrifugation. The viral titer was then assayed using an Adeno-X Rapid Titer kit (Clontech, Mountain View, CA).

### Cell culture

Two cell types were used in experiments: human umbilical vein endothelial cells (HUVECs) and human Schlemm’s Canal (SC) cells. HUVECs were obtained from Clonetics^TM^ and Lonza Walkersville, Inc. (Walkersville, MD). SC cells were isolated from human donor eyes, cultured and characterized as previously described (29). SC cell strains (passages 3–8) isolated from donors (SC73, SC69, SC89, SC91, SC78 and SC57, corresponding to ages of 37yo, 45yo, 68yo, 74yo, 77yo and 78yo at time of death, respectively) were used in experiments. HUVECs were cultured in Medium 199 (Gibco by Life Technologies, Grand Island, NY) supplemented with 15% fetal bovine serum (FBS, Atlanta Biological), penicillin-streptomycin-glutamine (PSG, 100 U/mL, Gibco by Life Technologies, Grand Island, NY), heparin sodium salt (90 μg/mL, Sigma-Aldrich, St. Louis, Mo) and endothelial mitogen (0.1 mg/mL, Biomedical Technology, Inc, Stoughton, MA). SC cells were cultured in DMEM Low Glucose 1X Medium (Gibco by Life Technologies, Grand Island, NY) supplemented with 10% FBS and PSG (100 U/mL). Cells were plated at confluence onto IbiTreat μ-slides I^0.6^ (Ibidi, Munich, Germany) and maintained for 3 days (HUVEC) or 7 days (SC) before transducing with adenovirus.

### Adenovirus transduction

To test upregulation of reporter proteins at different levels of shear stress, confluent cells in IbiTreat μ-slides I^0.6^ were transduced with either virus encoding eNOS promoter driving green fluorescent protein (eNOS-GFP) or eNOS promoter driving secretory alkaline phosphatase (eNOS-SEAP). Cytomegalovirus promoter driving GFP (CMV-GFP) was used as a shear-insensitive control (Supplemental Fig. 1). HUVEC and SC cells on μ-slides were transduced with 8.2 × 10^7^ virus particles. Both HUVEC and SC cells were exposed to the adenovirus for 8 hours before replacing the media with fresh media. After 48 hours, cells on μ-slides were exposed to shear stress of either low (0.1–1 dynes/cm^2^) or high (10–12.5 dynes/cm^2^) levels.

### Shear stress experiments

Shear stress was applied to confluent HUVEC and SC cells using an Ibidi pump system (Ibidi, Munich, Germany) as before. HUVEC and SC cells (3.3 × 10^5^ cells/slide) were seeded onto IbiTreat μ-slides I^0.6^ (Ibidi, Munich, Germany) and placed in an incubator at 37 °C with 5.0% CO_2_. HUVECs were allowed to settle for three days before induction of shear. In contrast, SC cells were allowed to settle for one to two weeks before a constant shear was applied. In a previous study^6^, we found this was the time required for cells to attach and maintain a stable monolayer during elevated shear stress. The Ibidi pump system (Ibidi, Munich, Germany) was set up as per the company’s instructions and proprietary software was used to control the level of shear applied to cells by controlling total media flow rate through the channels of known dimensions.

### Human donor eye tissues

Enucleated human eyes (10 pairs) were obtained from Miracles in Sight (Winston-Salem, NC) in accordance with the Declaration of Helsinki for research involving human tissue and with the Duke University Health System Institutional Review Board approval (protocol #PRO-00050810). According to approved protocols for the Miracles in Sight Eye Bank, informed consent was obtained from the next of kin for use of ocular tissues for research purposes. Enucleated eyes were free of any known ocular disease and were stored in a moist chamber at 4 °C until dissection and were used within 30 hours of eye donor’s death (Table 2). Eyes from glaucomatous donors were excluded from this study as they present with an increased incidence of collector channel blockage due to herniation of the trabecular meshwork which could prevent 360° transduction of the outflow pathway.

**Table 2:** Anterior segment donor and perfusion information.

Donor #	Age	Gender	TOD-TOE	TOD-TOP
760	69	M	6:08	23:10
1242	70	F	5:07	20:06
1371	71	M	6:15	12:17
1679	79	M	5:15	25:25
1784	53	M	6:15	29:50
2236	71	M	4:42	23:51
2580	70	M	6:24	33:14
2662	76	M	6:53	27:56
2677	79	M	4:56	30:44
339	60	M	4:16	30:21
**Mean**	**72.1**		**5:56**	**25:49**
**SD**	**8.27**		**0.04**	**6.17**

TOD – Time of death, TOE – Time of enucleation, TOP – Time of perfusion.

### Anterior segment dissection

Dissections were performed as previously described^48,49^. Briefly, the eyes were hemisected at the equator, and vitreous and lens were removed. The iris was trimmed from the root, and the vascular and pigmented tissues of the ciliary body were removed leaving the ciliary muscle and trabecular meshwork intact. The anterior segments were generously rinsed with media to remove cell debris and pigment.

### Anterior segment organ culture perfusions

The anterior segment perfusion model^48,49^ was used to study the eNOS-GFP and eNOS-SEAP adenoviruses. A syringe pump was used to generate a constant inflow rate for human eyes (PHD 2000 Programmable Syringe Pump, Harvard Apparatus, Holliston, MA). A wet-wet differential pressure transducer (0–50 mmHg; Omega Engineering, USA) continuously measured intrachamber pressure. Customized iPerfusion software^50^ recorded IOP measurements throughout the perfusion (multifunction DAQ, National Instruments, USA). A reservoir was connected between the perfusion chambers and the pressure transducer to facilitate media exchanges (DMEM with 4.5 g/L glucose, supplemented with 1% FBS, 1% PSG and 0.025% BSA) of perfusion chambers and to control intrachamber pressures for retroperfusion. When a stable baseline pressure was achieved at a constant inflow rate of 2.5 µl/min, the anterior segments were retroperfused with adenovirus. After the retroperfusion steps were completed, 2 protocols were followed after restarting forward perfusion. In all perfusions, paired anterior segments were initially perfused for 24–48 hours to achieve an initial stable baseline. In cases in which both segments achieved a stable baseline pressure (n = 5) forward perfusion was restarted as follows; in control anterior segments, a constant flow rate of 2.5 µl/min was maintained while in experimental anterior segments we increased the flow rate to 5 µl/min 24 hours post-retroperfusion. In cases where one of the anterior segments of a pair failed to establish a stable baseline pressure (n = 5), only the segment with a stable baseline was used for the experiment. In these cases, perfusion was initially restarted at 2.5 µl/min for 24 hours, before increasing the flow rate to 5 µl/min for 24 hours. Conditioned media was collected every 24 hours after retroperfusion and used for analysis of nitrite and SEAP content.

Once the perfusion was completed, the trabecular meshwork was removed to gain better visualisation of the outer wall of Schlemm’s canal, and the anterior segments were then fixed overnight in 3% PFA before being cut into quadrants for further analysis.

### Retroperfusion

We used a previously established retroperfusion method to facilitate direct access of the adenovirus reporters from the venous side into the Schlemm’s canal lumen of the anterior segments^51^. Briefly, after a stable baseline pressure within normal physiological limits was achieved, a small plastic strip coated with silicone grease on the lower edge was placed into the clamp ring of the perfusion chamber, creating a fluid-tight seal surrounding the limbus. Media containing both eNOS-SEAP and eNOS-GFP viruses at 2 × 10^6^ PFU/ml was loaded into this temporary reservoir to completely submerge the limbus, and intrachamber pressure was lowered to −1mmHg for 1 hour. During this time, the anterior segment chamber was placed on a small incline and rotated 90 degrees every 15 min. Intrachamber pressure was then maintained at 0 mmHg for an additional hour, and during this time, the intrachamber pressure was occasionally varied at ±1 mmHg for 15 s intervals to promote circumferential flow in the SC lumen. These pressure manipulations were imparted by moving a fluid reservoir connected to the anterior segment. Forward perfusion was then restarted according to the protocols outlined in the above section. In terms of the combination treatment of the anterior segments with both adenovirus reporters¸ we cannot assess relative transduction efficiency, but using GFP visualisation and SEAP output it is clear that both were successfully transduced. Given their similar size, identical virus background and identical titer we assumed approximately equal transduction efficiency.

### eNOS promoter activity: green fluorescent protein detection

#### Immunofluorescence microscopy

Cultured cells were imaged with a Nikon Eclipse 90i microscope with a Nikon D-Eclipse C1 Si Laser (Melville, NY). Identical gain and exposure settings were used to capture all images during a single viewing session. Five images were captured randomly along the approximate center line of the μ-slide, roughly equidistant from each other down the length of the slide. To assess GFP expression by cells in the SC lumen *in situ*, we removed the trabecular meshwork (TM) and inner wall of SC after perfusions, keeping the outer wall of SC intact. Anterior segments were divided into quadrants and non-specific sites were blocked with 3% bovine serum albumin (BSA, Sigma Aldrich, St. Louis, MO) and 3% normal goat serum (NGS, Sigma Aldrich, St. Louis, MO) in PBS with 0.1% Triton-X prior to incubation with a rabbit polyclonal IgG GFP antibody at 1:300 dilution. AlexaFluor 594 secondary antibody (Jackson Labs, USA) were used. A Nikon Eclipse Ti2 inverted confocal microscope (Nikon, Melville, NY) was used to obtain tile scans across the outer wall of SC, which were assembled and pseudo-coloured green in post-analysis using Image J.

### Collector channel analysis in ImageJ

To quantify GFP expression at locations of collector channel ostia in SC, images of *enface* SC outer walls were obtained as described above. Images were opened in ImageJ, clearly identifiable CC ostia were identified by an observer masked to the treatment group. All CCs were selected and completely encased in a consistent size square of 256 × 256 pixels. The mean grey value for each CC ostia was quantified, and all data were compiled prior to unmasking and subsequent analyses.

### Flow cytometry

Cells were removed from Ibidi plates at the conclusion of the shear stress experiments. Slides were rinsed with PBS before being incubated with 0.25% Trypsin-EDTA. Trypsinized cells were pelleted by centrifugation and resuspended in fresh PBS for analysis by flow cytometry to assess GFP expression (Foreessa SORP analyser, BD, Franklin Lakes, NJ). Briefly, cells pass single file in front of a 488 nm laser. Forward scatter, side scatter and the GFP intensities were all measured simultaneously using FACSDIVA 8 software (BD Franklin Lakes, NJ).

### Western blotting

Cells were collected by centrifugation and the cell pellet was resuspended in Laemmli sample buffer containing 10% β-mercaptoethanol. Whole cell lysates were boiled for 10 minutes, loaded onto 10% polyacrylamide gel, and proteins were fractioned by SDS-PAGE. Proteins were electrophoretically transferred from gel slabs to nitrocellulose membranes for 90 minutes at 100 V. Membranes were incubated overnight at 4 °C with either rabbit polyclonal IgG that recognizes GFP (1:3000 dilution) or rabbit polyclonal IgG that recognizes GAPDH (1:5000 dilution, Cell Signalling Technology, Danvers, MA). Blots were then incubated with horseradish peroxidase (HRP)-conjugated goat anti-rabbit (1:5000 dilution, Jackson Immunoresearch Laboratories, West Grove, PA). To visualize proteins, membranes were incubated with enhanced chemiluminescence reagents (GE Healthcare Life Sciences, Pittsburgh, PA) and imaged using a Biorad Chemidoc (Biorad, Hercules, CA).

### eNOS promoter activity: secreted alkaline phosphatase detection

Conditioned media collected from cell cultures and anterior segment perfusions (effluent) was assayed for placental alkaline phosphatase activity with the Tropix Phospha-Light System as recommended by the manufacturer (Applied Biosystems, Bedford, MA). Briefly, the samples were heated at 65 °C and then combined with a chemilumenescent alkaline phosphatase substrate prior to detection of sample chemiluminesence at 500 millisecond/well using a SpectraMax M5 plate reader coupled with SoftMax Pro 7 software (Molecular Devices, Sunnyvale, CA). A SEAP standard included in the assay was used to quantify SEAP content in experimental samples.

### Nitrite assay

Nitric oxide (NO) concentration was indirectly assessed by measuring the concentration of nitrite, a by-product of NO degradation, with a Measure-IT™ High-Sensitivity Nitrite Assay Kit according to the manufacturer’s directions (Invitrogen by Life Technologies, Grand Island, NY). Conditioned media collected from flow chambers and the anterior segment perfusions every 24 hours was used to measure nitrite concentration in the samples. Briefly, a standard curve was made using serial dilutions of a nitrite standard included in the kit. The samples and standards were combined with a working solution included in the kit and incubated at room temperature. The fluorescence of the nitrite-containing samples was measured at using a SpectraMax M5 plate reader coupled with SoftMax Pro 7 software (Molecular Devices, Sunnyvale, CA). Linear regression analysis using data from the standard curve was used to estimate the nitrite concentrations of the samples.

### Statistical analyses

Data are presented as mean ± standard deviation. The Student’s *t*-test was used to analyse for statistical significance (P < 0.05). “n” indicated in results or figure legends refers to independent experiments (HUVECs and SC cells) and tissue from independent donors. Outliers (>2 standard deviations outside of the mean) were not included in the final analyses.

## Supplementary information

Supplemental Info.
