# Supplementary material for: Shear Stress in Schlemm’s Canal as a Sensor of Intraocular Pressure
Source: Sci Rep. 2020 Apr 2;10:5804. doi: 10.1038/s41598-020-62730-4 (PMC7118084; doi:10.1038/s41598-020-62730-4)
Supplement: Supplementary file 1 — Supplemental Info. [file 41598_2020_62730_MOESM1_ESM.docx]

**Shear Stress in Schlemm’s Canal as a Sensor of Intraocular Pressure**

Fiona McDonnell^1^, Kristin M. Perkumas^1^, Nicole E. Ashpole^1^, Joan Kalnitsky^1^, Joseph M. Sherwood^2^, Darryl R. Overby^2^, W. Daniel Stamer^1,3*^

1. Duke Eye Center, Durham, North Carolina, USA.

2. Bioengineering, Imperial College London, London, UK.

3. Biomedical Engineering, Duke University, Durham, USA.

* Corresponding Author: W. Daniel Stamer, Duke University, DUMC 3802, Durham, NC 27710,

USA

[dan.stamer@duke.edu](mailto:dan.stamer@duke.edu)


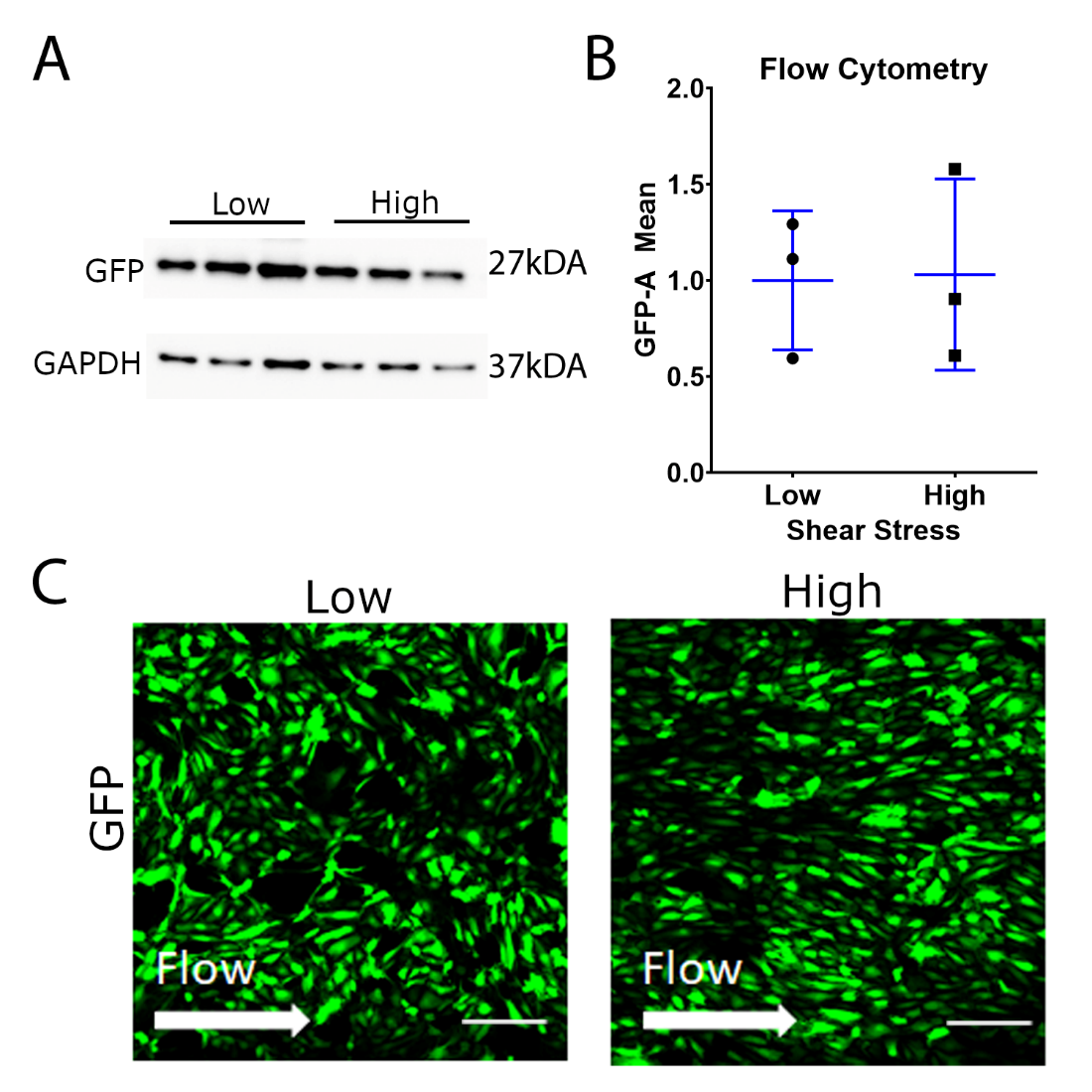


**Supplemental Figure 1: GFP expression in HUVECs transduced with shear-insensitive CMV-GFP adenovirus.** HUVECs were transduced with either CMV-GFP adenovirus and subjected to low or high shear stress for 24 hours. Cellular GFP expression was measured using (A) Western blotting (B) flow cytometry and (C) confocal microscopy**.** This demonstrated no change in GFP expression under different levels of shear stress. Full length Western blots shown in Supplemental Figure 3A. Scale bar - 50µm

**
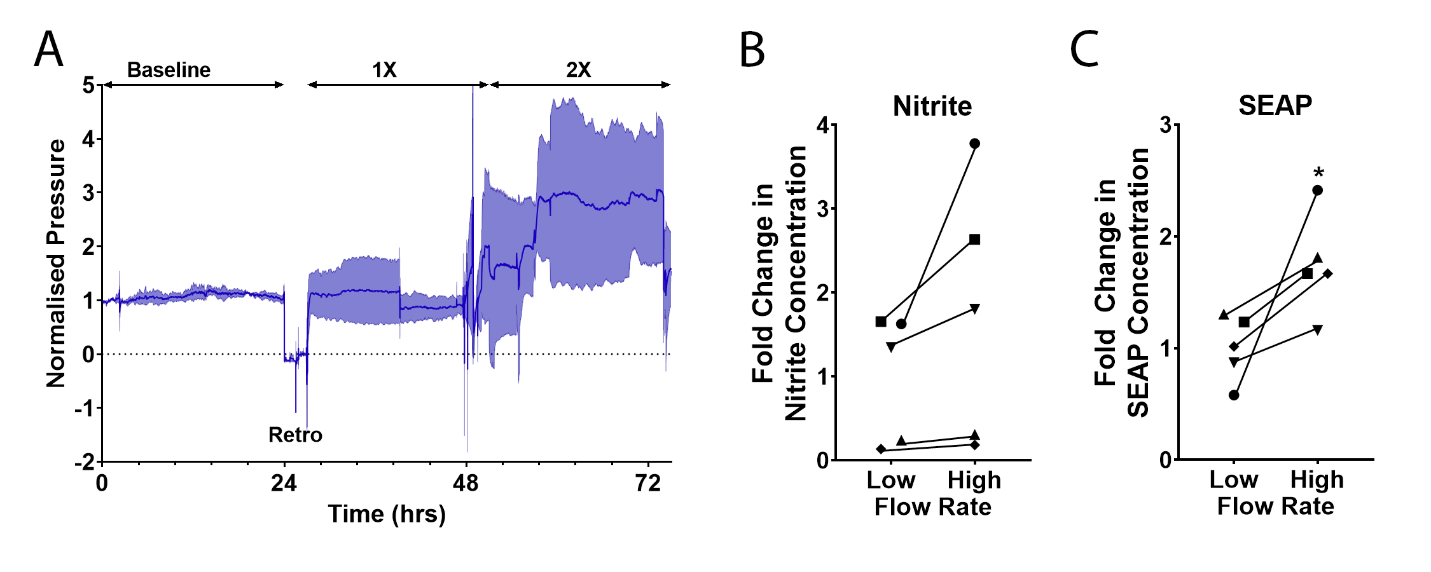
**

**Supplemental Figure 2. Single human anterior segment retroperfused with adenovirus reporters.** Single segments were perfused at a constant flow rate of 2.5 µl/min to achieve a stable baseline. Following this, a cocktail of both eNOS-SEAP and eNOS-GFP viruses was retroperfused into the anterior segment. The flow rate was maintained at 2.5 µl/min for 24 hours, then increased to 5 µl/min for 24 hours. Effluent media was then collected every 12 hours for analyses. 72 hours after retroperfusion, segments were fixed in 3% paraformaldehyde for further analyses. (A) Perfusion trace showing manipulations throughout perfusion, solid line represents mean, shaded areas show standard deviation. Effluent was collected every 24 hours and (B) Nitrite concentration by nitrite quantification assay and (C) SEAP concentration was measured using a chemiluminescent SEAP assay. Samples were normalized to volume. n=5, *P<0.05, 1X = 2.5 µl/min, 2X = 5µl/min flow rates.


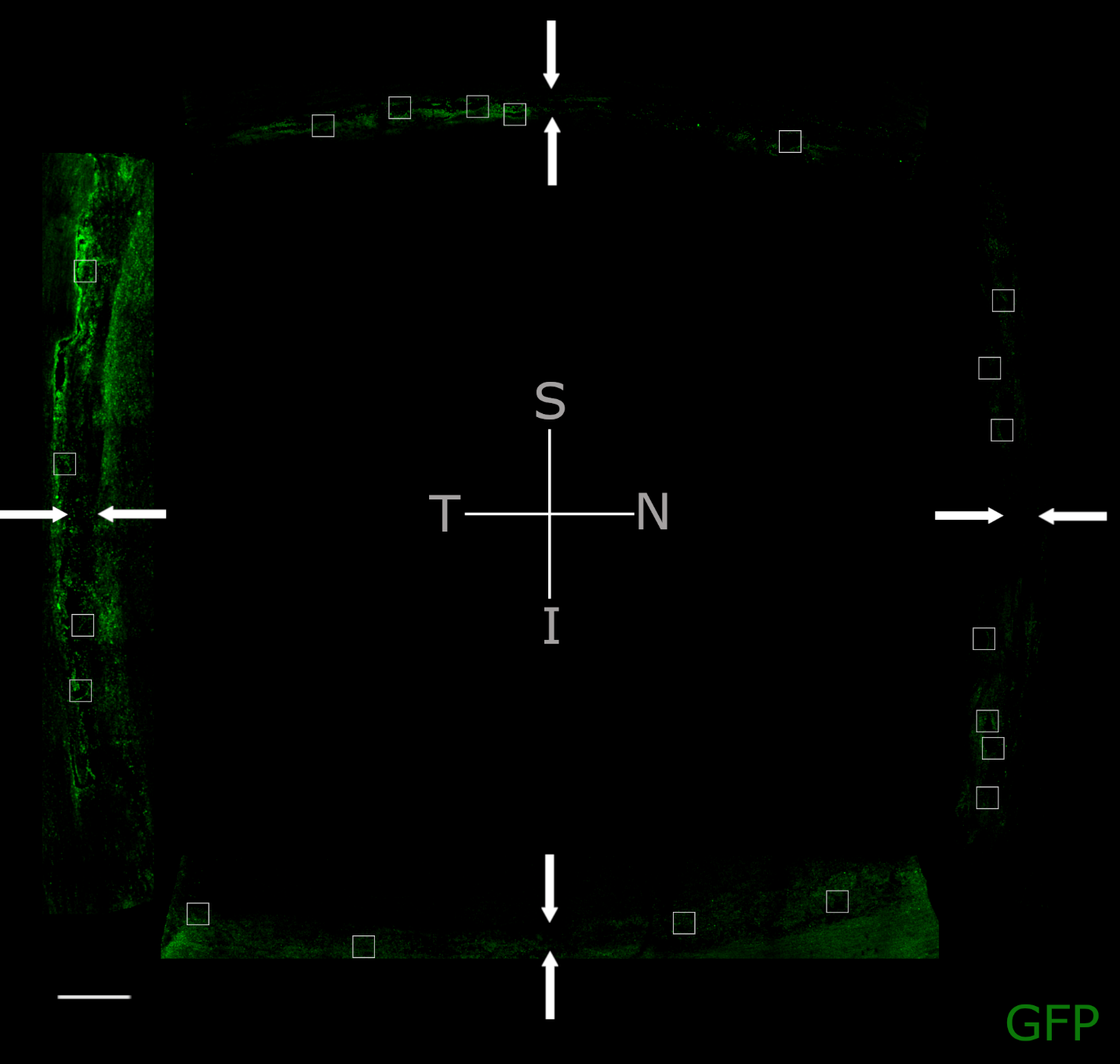


**Supplemental Figure 3: GFP expression in unpaired anterior segments retroperfused with adenovirus reporters.** Representative human anterior segments that was perfused at a constant flow rate of 2.5 µl/min to achieve a stable baseline. Once achieved, a cocktail of both eNOS-SEAP and eNOS-GFP viruses was retroperfused into Schlemm’s canal. 24 hours post-retroperfusion, the flow rate in one segment was then increased for 24 hours. 72 hours after retroperfusion, segments were fixed in 3% paraformaldehyde for further analyses. Immunofluorescence showing GFP expression across the outer wall of SC. Boxes highlight identifiable collector channel ostia. Scale Bar - 500µm

**
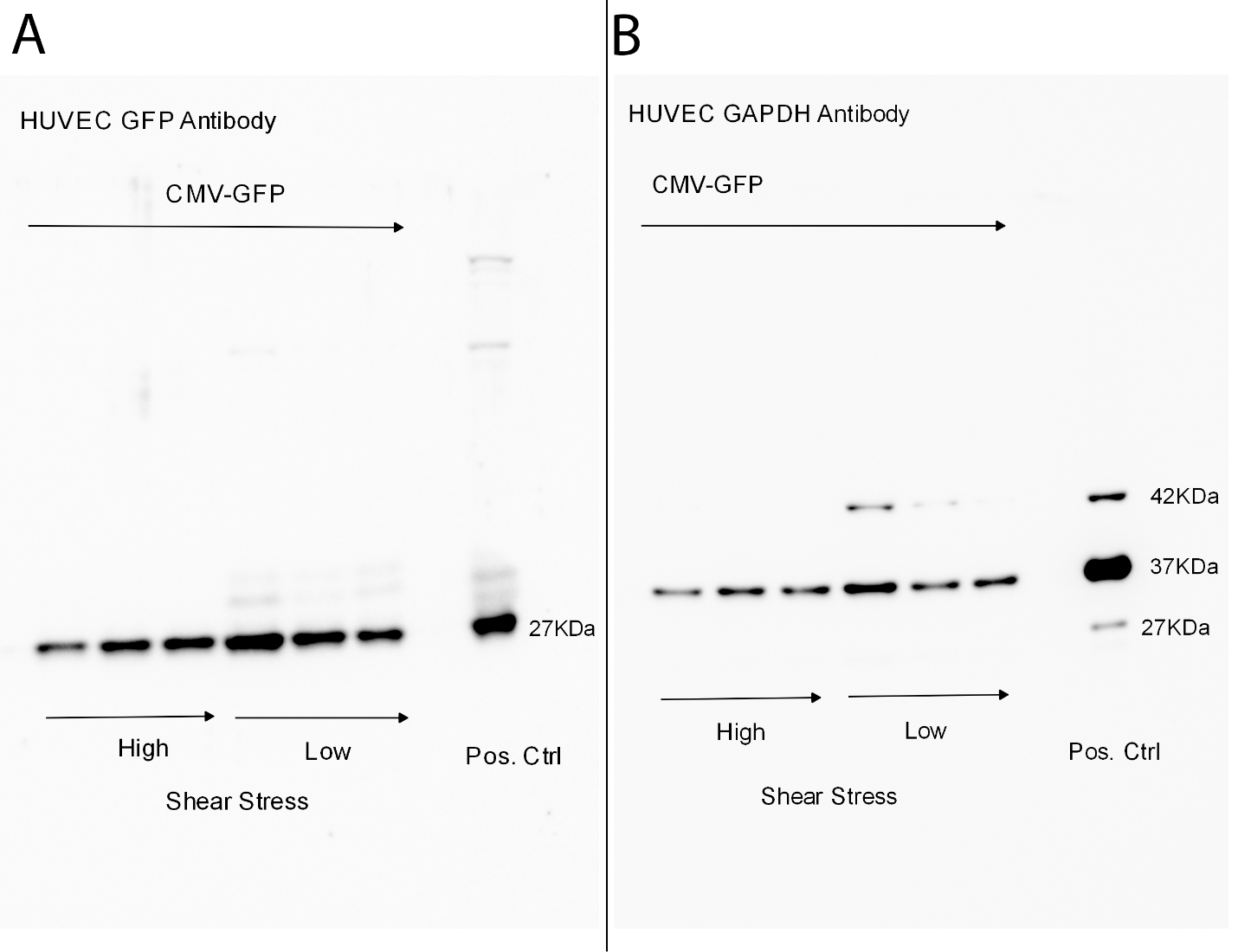
**

**Supplemental Figure 4: Full length Western blots**

Full length Western blot for cropped blots shown in Supplemental Figure 1A. Pos. Ctrl = Positive Control

| **Pressure (mmHg)** | **Baseline** | **1X** | **2X** |
| --- | --- | --- | --- |
| **Single Anterior Segment Perfusions** | 11.32±2.151 | 11.11±1.077 | 22.19±7.997 |
| **Paired Anterior Segment Perfusions – Low Flow Segment** | 9.109±3.922 | 9.050±1.114 | NA |
| **Paired Anterior Segment Perfusions – High Flow Segment** | 11.03±2.763 | 12.82±2.456 | 33.13±14.21 |

Supplemental Table 1: Average intrachamber pressure data from anterior segment perfusions. Data presented as mean±SD, 1X = 2.5 µl/min, 2X = 5µl/min flow rates.

| **Nitrite Concentration (µM)** | **1X** | **2X** |
| --- | --- | --- |
| **Single Anterior Segment Perfusions** | 0.99±0.75 | 1.73±1.52 |
| **Paired Anterior Segment Perfusions – Low Flow Segment** | 9.79±11.19  5.49±2.99  6.43±3.15 | NA |
| **Paired Anterior Segment Perfusions – High Flow Segment** | NA | 17.44±17.53  7.66±3.70  10.02±6.98 |

Supplemental Table 2: Raw nitrite concentrations (µM) for anterior segment perfusions. Data presented as mean±SD, 1X = 2.5 µl/min, 2X = 5µl/min flow rates.
